# The Importance of H in Particulate Organic Matter Stoichiometry, Export and Energy Flow

**DOI:** 10.3389/fmicb.2017.00826

**Published:** 2017-05-09

**Authors:** David M. Karl, Eric Grabowski

**Affiliations:** Daniel K. Inouye Center for Microbial Oceanography: Research and Education, Department of Oceanography, School of Ocean and Earth Science and Technology, University of Hawaii, HonoluluHI, USA

**Keywords:** hydrogen, energy, organic matter, particle flux, stoichiometry

## Abstract

The discipline of marine ecological stoichiometry has progressed rapidly over the past two decades, and continues to be at the forefront of microbial oceanography. Most of this effort has been focused on the elements carbon (C) and nitrogen (N), and to a lesser extent phosphorus (P), with little consideration of hydrogen (H), or the redox state of the organic matter pools despite the fact that H is the most abundant, and possibly the most important, element in biogeochemistry. Obtaining accurate estimates of the H content of organic matter, either in suspended or sinking particles, is a major analytical challenge. While many aquatic science laboratories have access to commercial “C–H–N elemental analyzers,” few investigators report H values due to analytical difficulties in obtaining accurate estimates of H. Because organic compounds vary considerably in their H:C ratio and therefore in their energy content, measurements of H combined with C-specific caloric estimates will ultimately be required for a more comprehensive understanding of ecosystem dynamics.

## Introduction

More than one century ago, in his authoritative treatise on *Conditions of Life in the Sea*, James Johnstone stated that “chemical analysis shows that the animal and plant body is mainly built up from four elements: nitrogen (N), carbon (C), hydrogen (H), and oxygen (O). Added to these are the metals sodium, potassium, and iron, and the non-metals chlorine, sulfur (S), and phosphorus (P)” (Johnstone, 1908). He went on to conclude “that in an exhaustive study of the cycle of matter from the living to the non-living phases, and vice versa, we should have to trace the course of each.”

Pioneering research conducted by Alfred C. Redfield on the composition of marine plankton, which first appeared in the James Johnstone Memorial Volume, established a quantitative relationship between dissolved nitrate and phosphate, and between dissolved nitrate and total carbon dioxide in water column profiles from the western North Atlantic Ocean. The mean molar changes with depth, expressed as ΔN:ΔP and ΔC:ΔN ratios, were 20:1 and 7:1, respectively, or a C:N:P molar ratio of approximately 140:20:1 (Redfield, 1934). Furthermore, the molar ratio of dissolved oxygen (O_2_) decrease to dissolved nitrate increase was 6:1, suggesting that microbial decomposition of organic matter was the ultimate source of nitrate in the mesopelagic zone (400–1,000 m) of the ocean (Redfield, 1934). Furthermore, elementary analysis of naturally occurring plankton samples yielded proportions of C, N, and P that were “not greatly different” from those observed in oceanic waters. Redfield lamented that this exact balance of N and P, two major plant nutrients, “calls for some explanation” since it would appear that it was more than a coincidence (Redfield, 1934). Several potential mechanisms were presented to explain what Redfield termed “a phenomenon of the greatest interest” thereby setting the stage for the ecological stoichiometry revolution that was to follow (Sterner and Elser, 2002).

Subsequent research by Redfield and many others led to a more comprehensive understanding of biological control of nutrient distributions in the sea, and the structure and pace of biogeochemical cycles. Redfield (1958) also laid the foundation for what was later dubbed the biological pump. The main components included: (1) photosynthetic production of organic matter in the upper 0–200 m of the water column, (2) loss of a small portion of the newly synthesized organic matter by the combined effects of passive sinking and active vertical migration, and (3) decomposition of organic matter at depth to regenerate inorganic nutrients. Both the net production and net remineralization of organic matter resulted in changes in the concentrations of dissolved inorganic C, N, and P, and the quantity of O_2_ required for complete oxidation reflected both the elemental composition and the redox state of the major biogenic constituents. This quantitative analysis of photosynthesis and respiration, including restoration of the N:P ratio via N_2_ fixation and extension to anaerobic sulfate reduction processes in selected regions where the resupply of O_2_ was restricted, established the unifying principle that we now commonly refer to as “the Redfield–Ketchum–Richards (R–K–R) Ratio” (Redfield et al., 1963).

## Elemental Composition of Marine Phytoplankton

The R–K–R model assumes that marine phytoplankton have an average molar composition of C_106_H_263_O_110_N_16_P, and further assumes that the complete oxidation of this model organic matter following cell death would consume 138 molecules of O_2_. These values, or slight variations of this elemental stoichiometry (see below), have been used for nearly 50 years to model biogeochemical processes in the sea (Sarmiento and Gruber, 2006). However, the amount of O_2_ consumed during complete microbial remineralization of organic matter depends critically on its molecular composition, specifically the bulk H:C ratio, the S content and the presence or absence of reduced storage materials. Furthermore, no previous study of ecological stoichiometry has considered the possible presence of reduced P (valence states < +5) which is now established as a ubiquitous constituent of marine organic matter (Karl, 2014; Van Mooy et al., 2015; Repeta et al., 2016). Finally, for mass balance consideration of ecological stoichiometry and reduction–oxidation processes, one also needs to consider dissolved organic matter (DOM), reduced gases (e.g., H_2_, N_2_O, CO, CH_4_, dimethyl sulfide (DMS)/H_2_S) and reduced inorganic compounds that may be produced and released during photosynthesis, and that ultimately contribute to the demand for O_2_ during subsequent microbial oxidation. For example, during the process of N_2_ fixation, H_2_ is produced in stoichiometric proportion to N_2_ reduced. A variable portion of this H_2_ is lost from the cell, along with ammonia and dissolved organic N. These reduced compounds are in excess of those required for the synthesis of new plankton biomass and should be considered for an accurate accounting of ecological stoichiometry, biochemical O_2_ demand and energy flow in marine systems.

All living organisms are comprised of basic macromolecules including carbohydrate, lipid, protein, and nucleic acid, each with a unique bulk elemental stoichiometry and energy content. Direct analysis of the macromolecular composition of sample material, a so-called proximate analysis, has been used to estimate the energy content of organic matter by the application of class-specific conversion factors (e.g., 9.45 calories mg^-1^ for lipid, 5.65 calories mg^-1^ for protein; Craig et al., 1978). This approach fails to account for all possible organic and reduced inorganic compounds, and is probably more accurately applied to pure cultures or tissues than to naturally occurring dissolved and particulate organic matter (POM). Since the H:C and O:C ratios among these macromolecular classes vary considerably (Laws, 1991), the theoretical amount of reducing potential required for biosynthesis and of O_2_ consumed for complete oxidation following death (generally expressed as the -O_2_:P ratio; see below) will also vary. Based on considerations of the proximate analysis of typical marine phytoplankton cells, Anderson (1995) concluded that the R–K–R elemental stoichiometry (i.e., C_106_H_263_O_110_N_16_P) was too high in both H and O, relative to P. He proposed a new formulation, C_106_H_175_ ± _11_O_42_ ±_16_N_16_P with a ΔO_2_: ΔP ratio of -150 ± 19, as a more accurate representation. The H and O contents for this revised elemental stoichiometry are 32–35% and 50–74%, respectively, lower than the R–K–R model plankton (Anderson, 1995).

Platt and Irwin (1972) reported a partial proximate analysis (i.e., carbohydrates, protein, and lipid, but did not include nucleic acids or storage products), the bulk C:N ratios and caloric content (measured directly using a Phillipson microbomb calorimeter; Phillipson, 1964) for “phytoplankton” samples (76–153 μm size fraction) collected during the spring bloom (April 1969) in St. Margaret’s Bay, Nova Scotia. This comprehensive, 1-month field study revealed large day-to-day variations in percentage composition of major macromolecular classes [relative to total dry weight (dw)]. For example, the % carbohydrate:% protein ratios ranged from 0.56 to 2.45, and % carbohydrate:% lipid ratios ranged from 0.74 to 8.9, while bulk C:N molar ratios ranged from 5.5 to 17.2. Despite this time-variable composition, they observed a much smaller range in the C-specific caloric content from 10.07 to 13.66 calories mg^-1^ C. Platt and Irwin (1972) concluded that “percent carbon in dry phytoplankton should be a good predictor of its caloric value,” and presented the following relationships for general use in marine ecology: calories (mg dw^-1^) = 0.632 + 0.086 (%C), and if N content is also known: calories (mg dw^-1^) = -0.555 + 0.113 (%C) + 0.054 (C:N).

The approach proposed by Platt and Irwin (1972) was developed for the phytoplankton-enriched portion of the spring bloom where most of the POM pool was comprised of living cells. However, in most ecosystems the organic matter pool is dominated (>90% by mass) by DOM, not POM, and the large, chemically complex DOM pool may have a molecular composition and elemental stoichiometry that is different from living cells. For example, whereas the contributions of amino acids, neutral sugars, and lipids together account for >80% of the organic C content of phytoplankton, they contribute <10% of the total C in DOM (Benner, 2002). Although the molecular composition of DOM is poorly known, significant progress has been made in recent years using several DOM extraction methods and a variety of high resolution analytical techniques (Repeta, 2015). For open ocean environments, the H:C and O:C ratios of DOM collected by solid-phase extraction (SPE) of DOM onto polymeric resins (e.g., Amberlite^®^ XAD^®^), range from 0.5 to 1.7 and 0.2 to 0.8, respectively, and are mostly outside the range of proteins, carbohydrates, and lipids, suggesting that SPE-DOM is either extensively degraded relative to plankton or has some other non-marine source (Repeta, 2015). Humic substances, operationally defined as SPE-DOM that is retained on XAD-2 or XAD-8 resins under acidic conditions (Benner, 2002), are ubiquitous in marine environments and appear to be resistant to microbial decomposition. However, selected microorganisms can use humic substances as electron acceptors during anaerobic metabolism, thereby facilitating the degradation of organic matter (Lovley et al., 1996).

Furthermore, in most oligotrophic environments, a majority of the POM pool is non-living (Karl and Dobbs, 1998), so it too may be chemically distinct from the elemental composition of biomass. Lee et al. (2004) have shown that the preferential removal of certain components from POM leads to changes in the chemical composition of the residual material as particles sink through the water column. Compared to surface ocean plankton where ∼85% of total C can be chemically identified, the majority of POM collected in a sediment trap deployed at a 1,000 m reference depth could not be characterized. Consequently, models of the molecular composition of organic matter that are based on living cells may not accurately reflect organic matter that is present in natural ecosystems. Compared to a wealth of field data on particulate organic C:N:P, and a much smaller data base on dissolved organic C:N:P, there is a virtual absence (with very few notable exceptions; see below) of direct measurements of the H, O, S, or total caloric content of either the POM or DOM pools. This situation exists despite the central role of H in stoichiometry and energy flow in marine ecosystems.

## Remineralization Ratios in the Open Sea

Several field studies, beginning with Takahashi et al. (1985), have measured concentrations of phosphate, nitrate, total carbon dioxide, and O_2_ along deep isopycnal horizons to constrain the elemental composition of the organic matter that supplied the deep sea with excess regenerated nutrients via aerobic microbial activity. For intermediate waters of the North Atlantic Ocean (σ_𝜃_ = 27.0–27.2), their analysis indicated that the C:P and N:P ratios were not significantly different from the remineralization of model R–K–R plankton. However, the mean -O_2_:P ratio of 171 ± 8 was much larger than that predicted from either the remineralization of R–K–R or Anderson (1995) model plankton (e.g., 138 and 150, respectively). They further concluded that the decomposing organic matter was more accurately represented by the chemical reaction, C_2_H_4_ + 3O_2_ → 2CO_2_ + 2H_2_O, rather than the oxidation of CH_2_O. They emphasized the importance, but also acknowledged the absence, of quantitative knowledge of the H content of the organic matter (Takahashi et al., 1985). Similar results of higher than predicted -O_2_:P ratios were also reported by Broecker et al. (1985), Minster and Boulahdid (1987), Peng and Broecker (1987), Boulahdid and Minster (1989), and Anderson and Sarmiento (1994), all in excess of -170:1. These extensive field results are consistent with the oxidation of organic matter that is on average more reduced than carbohydrate (CH_2_O), the assumed oxidation state in R–K–R model plankton. Alternatively, yet unidentified reduced constituents contained in marine organic matter (e.g., reduced P; Karl, 2014) might also contribute to the demand for O_2_ during microbial decomposition.

## Station Aloha: a Case Study

Station ALOHA (22°45′N, 158°W) was established in October 1988 as a strategically located oligotrophic ocean benchmark in the North Pacific Subtropical Gyre (NPSG) to improve our understanding of microbial and biogeochemical processes that control carbon and energy fluxes in the upper kilometer of the ocean (Karl and Lukas, 1996). On approximately monthly intervals since the establishment of Station ALOHA, samples have been collected for a host of physical, chemical, and biological measurements including several key features of the biological pump (Karl and Church, 2014, 2017). The C:N:P of both suspended (Hebel and Karl, 2001) and sinking (Karl et al., 1996) particulate matter pools, and the N:P of the DOM pool (Karl et al., 2001) have revealed complex, time-variable interactions between the dynamics of N and P, and unexpected temporal variability in stoichiometry, in addition to secular trends (also see Karl, 2014).

Li et al. (2000) developed a simple mixing model of two end-members to estimate the organic matter remineralization ratios at Station ALOHA. Based on their analysis of data collected during 1994, and assuming a H:C ratio of 1.65 for marine phytoplankton (Anderson, 1995), the molar formula of remineralized organic matter was C_135_H_280_O_105_N_13_P or C_25_(CH_2_O)_101_(CH_4_)_9_(NH_3_)_13_(H_3_PO_4_). Complete oxidation of this hypothetical organic matter would consume 170 O_2_ (i.e., -O_2_:P = -170), and yield 135 CO_2_ + 132 H_2_O + 13 NO_3_^-^ + 1 H_2_PO_4_^-^ + 14 H^+^ (Li et al., 2000). These results were consistent with the Takahashi et al. (1985) observations and indicative of a more reduced organic matter source than either R–K–R or Anderson (1995) model plankton.

Two pioneering sediment trap experiments reported organic C, H, and N data for sinking particles collected in the NPSG (Honjo, 1980; Martin et al., 1987). We have recently refined the analytical procedures necessary for the routine estimation of the H in sinking particulate matter and, herein, add a third NPSG data set that we compare and contrast to the pioneering efforts of Honjo (1980) and Martin et al. (1987).

Honjo (1980) reported depth-variable molar H:C ratios for POM samples collected in an array of five bottom-moored sediment traps positioned at depths between 378 and 5,582 m (**Table 1**). Although the bulk molar H:C ratio was highest at the shallowest reference depth sampled (e.g., 1.79 at 378 m) and was lower at greater depths (molar H:C ranged from 1.50 to 1.64), there was no systematic change with depth despite a large decrease in the flux of total organic C from 3.56 mg C m^-2^ d^-1^ at 378 m to 0.66 mg C m^-2^ d^-1^ at 5,582 m (**Table 1**). These observations suggest that there are only relatively small changes in the mean redox state of organic matter as particles sink through the water column despite a loss of >80% of organic matter mass over this same depth interval. These results are more consistent with a physical (disaggregation/dissolution) model of particle attrition with increasing water depth than with biochemical (microbial degradation) control. The latter might be expected to selectively remove more reduced, energy-rich organics during the decomposition process. Alternatively, these results could be explained by the presence of recalcitrant (or semi-labile) reduced organic matter that buffers the bulk H:C molar ratio in sinking particles. Honjo (1980) also reported the elemental composition of different size fractions (>1 mm, 63 μm – 1 mm and <63 μm) of the collected organic matter. Although the percentage of the total organic matter collected in these various size fractions changed systematically with depth, with a shift toward smaller particles in deeper waters, the molar H:C ratios did not track the particle size distributions, and overall exhibited much greater variability than the values reported for the elemental composition of the “total” POM pool (presumably the mass-weighted mean value; Honjo, 1980). This comprehensive study by Honjo (1980) also included sediment trap data sets from sites in the Sargasso Sea (two reference depths) and the equatorial Atlantic Ocean (four reference depths). For samples collected in the equatorial Atlantic Ocean, the molar H:C ratios systematically increased from 1.44 at 389 m to 2.75 at 5,068 m. Honjo (1980) commented that these unusually high deep ocean H values, and depth-dependent increases in %H of the organic matter and molar H:C ratios “may be partly due to the evaporation of structural or pseudostructural water from opal and clay particles” and, therefore, may not accurately reflect the H contents of POM. Despite these limitations and uncertainties, the report by Honjo (1980) stands as a benchmark study of the elemental stoichiometry of exported particles even 35 years after it was published. Getting good H data in field samples continues to be a significant analytical challenge.

**Table 1:** Elemental analysis of sinking particulate organic matter collected in the North Pacific Subtropical Gyre.

Study location, water depth (m), and reference	Trap depth (m)	C flux (mg m^-2^ d^-1^)	H flux (mg m^-2^ d^-1^)	N flux (mg m^-2^ d^-1^)	H:C (molar)	C:N (molar)
15°21′N, 151°29′W, 5,792 m (Honjo, 1980)	378	3.56	0.53	0.46	1.79	9.00
	978	0.55	0.07	0.07	1.53	9.17
	2,778	1.09	0.14	0.12	1.54	10.6
	4,280	0.88	0.11	0.10	1.50	10.3
	5,582	0.66	0.09	0.08	1.64	9.62
28°N, 155°W, 5,000 m (Martin et al., 1987)	100^∗^	38.5	4.05	6.41	1.26	6.99
	200	19.6	2.20	2.97	1.35	7.70
	300	13.2	1.54	1.89	1.40	8.15
	500	8.04	0.97	1.07	1.45	8.77
	750	5.42	0.68	0.68	1.51	9.30
	1,000	4.10	0.53	0.50	1.55	9.52
	1,500	2.76	0.37	0.32	1.61	10.1
	2,000	2.09	0.29	0.23	1.67	10.6
22°45′N, 158°W, Station ALOHA, 4,850 m (This study)	100	43.8 ± 0.6	6.11 ± 0.04	7.10 ± 0.08	1.66	7.21
	110	46.8 ± 0.9	6.40 ± 0.08	7.42 ± 0.14	1.63	7.36
	120	51.7 ± 1.6	6.93 ± 0.24	8.19 ± 0.50	1.60	7.37
	130	33.0 ± 0.9	4.24 ± 0.18	4.70 ± 0.29	1.53	8.19
	140	33.8 ± 0.2	4.22 ± 0.03	5.03 ± 0.10	1.49	7.83
	150	34.2 ± 1.1	4.25 ± 0.17	4.76 ± 0.27	1.48	8.40
	160	41.6 ± 2.1	5.17 ± 0.32	6.09 ± 0.45	1.48	7.97
	175	34.3 ± 0.3	4.19 ± 0.04	4.54 ± 0.08	1.46	8.81
	200	27.9 ± 0.5	3.31 ± 0.06	3.61 ± 0.05	1.42	9.00
	250	26.0 ± 0.7	3.10 ± 0.08	3.13 ± 0.19	1.42	9.69
	300	19.36 ± 1.0	2.25 ± 0.14	2.16 ± 0.13	1.38	10.5

*^∗^Values for the reference depths presented were calculated from the normalized power function [F = F_100_ (z/100)^b^] for the VERTEX 5 data set.*

The second major study of bioelemental cycling in the NPSG was conducted as part of the decade-long VERtical Transport and EXchange (VERTEX) program that included observations at 28°, 155°W during summer 1983 (Martin et al., 1987). Measurements of C, H, and N in sinking POM were made at nine separate depths between 50 and 2,000 m using a free-drifting array of sediment traps. This experiment provided estimates of element-specific fluxes, as well as remineralization rates based on changes in element fluxes versus water depth (**Table 1**). The trap-derived flux data were analyzed using a log–log transformation and a normalized power function, *F_z_* = *F*_100_ (*z*/100)*^b^*, where *F_z_* is the flux at any depth (*z*), *F*_100_ is the log–log intersect (also equivalent to the flux at 100 m), *z* is the depth in meters and the exponent *b* is the log–log slope of the flux versus depth relationship (also defines the flux attrition with depth). For samples collected in the NPSG, the attrition exponents (b) for C, H, and N were -0.973 (*r*^2^ = 0.95), -0.883 (*r*^2^ = 0.91), and -1.110 (*r*^2^ = 0.95), respectively, suggesting that the average composition of sinking particles becomes N-depleted and H-enriched, relative to C, as particles sink/age. At the 2,000 m reference depth, the molar C:N and H:C ratios were 10.6 and 1.67 compared to 6.99 and 1.26 at the 100 m reference depth (see **Table 1**). These observations contradict the results of Honjo (1980), who failed to show any systematic change in either C:N or H:C as a function of water depth for sediment-trap collected particles in the NPSG (**Table 1**). The Martin et al. (1987) observations of an increase in the molar H:C ratio in bulk organic matter as a function of depth/age, might be explained by the presence of a pool of recalcitrant (or semi-labile), reduced organic matter that resists microbial decomposition, though we have no direct evidence for any such pool. Furthermore, this trend of increasing H:C with depth was not seen in the Honjo (1980) data, even though sinking particulate matter was sampled over a much greater depth range (378–5,582 m).

**FIGURE 1:**
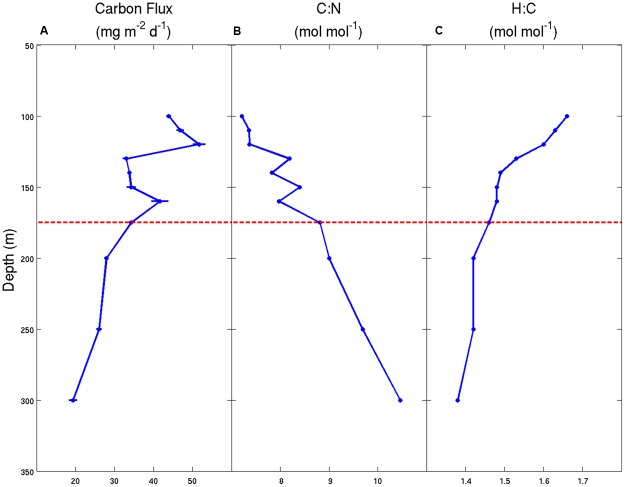
**Carbon flux and stoichiometry of particulate matter for samples collected using sediment traps deployed in the NPSG during the Hawaii Ocean Experiment-Dynamics of Light And Nutrients (HOE-DYLAN) IX expedition (August to September 2012). (A)** Particulate C flux (mg C m^-2^ d^-1^) ± 1 standard deviation of the mean (*n* = 3); **(B)** molar C:N ratios of the total particulate matter pool; **(C)** molar H:C ratios of the total particulate matter pool. The horizontal dashed line at 175 m represents the approximate location of the photosynthetic compensation depth where net photosynthesis is equal to 0 over a 24-h period.

Similar to the sediment trap data sets of Honjo (1980) and Martin et al. (1987), our observations at Station ALOHA also reveal significant and systematic decreases in the fluxes of C, H, and N with increasing water depth (**Table 1**). Over a fairly narrow depth interval (100–300 m), the fluxes of all three bioelements decreased by 60–70% relative to the maximum fluxes observed in the 100–120 m depth stratum. This rapid loss of sinking particles is a manifestation of the combined effects of particle dissolution, disaggregation, consumption by metazoans and microbial decomposition. Though we lack quantitative information on the primary controlling mechanism(s), we hypothesize that the two latter processes are most important in the upper mesopelagic zone. Furthermore, our data suggest that sinking particles become more C-enriched, relative to N and H, with increasing water depth (**Table 1** and **Figure 1**). The molar H:C ratios decreased from 1.66 at 100 m to 1.38 at 300 m, indicating the selective removal of organic matter that had, on average, a molar H:C ratio of approximately 1.9 based on the ΔH:ΔC observed over this depth interval. This value is nearly identical to the H:C ratio in monosaccharides (e.g., C(H_2_O)n; H:C = 2.0) and to the C_2_H_4_ substrate hypothesized by Takahashi et al. (1985). Without direct estimates of the O or total caloric content of the sinking particles, we are not able to further characterize the mean particle redox state or energy content, respectively. However, it appears almost certain that particles become more oxidized, and have a lower C-specific energy content and a lower C-specific biochemical O_2_ demand for complete oxidation as they sink/age. These lower energy particles are less desirable substrates for zooplankton and bacteria, and are able to escape the remineralization-intensive zone of the upper mesopelagic and continue their journey toward the abyss.

Most current models of C and energy flow through planktonic marine ecosystems do not include an explicit consideration of redox state or energy content of organic matter or how it varies during the decomposition process or as particles sink/age. Measurements of H help to constrain these processes and add a new dimension to ecosystem dynamics and the controls on the biological C pump. Consideration of the H in stoichiometry, in our opinion, is long overdue.

## Author Contributions

DK designed the study. EG provided data and interpretation. DK and EG wrote the paper.

## Conflict of Interest Statement

The authors declare that the research was conducted in the absence of any commercial or financial relationships that could be construed as a potential conflict of interest.
